# A 0.8 V, 14.76 nVrms, Multiplexer-Based AFE for Wearable Devices Using 45 nm CMOS Techniques

**DOI:** 10.3390/mi14101816

**Published:** 2023-09-23

**Authors:** Esther Tamilarasan, Gracia Nirmala Rani Duraisamy, Muthu Kumaran Elangovan, Arun Samuel Thankmony Sarasam

**Affiliations:** 1 Department of Electronics and Communication, Thigarajar College of Engineering, Madurai 625005, Tamil Nadu, India; 2 Engineering, D.R.B.R. Ambedkar Institute of Technology, Port Blair 744101, Union Territory of Andaman & Nicobar Islands, India; reachemk@gmail.com; 3 Department of Electronics and Communication , National Engineering College, Kovilpatti 628503, Tamil Nadu, India; arunsamuelece@nec.edu.in

**Keywords:** wearable medical devices (WMD), analog front end (AFE), operational transconductance amplifier, analog multiplexer, CMOS, Cadence Virtuoso

## Abstract

Wearable medical devices (WMDs) that continuously monitor health conditions enable people to stay healthy in everyday situations. A wristband is a monitoring format that can measure bioelectric signals. The main part of a wearable device is its analog front end (AFE). Wearables have issues such as low reliability, high power consumption, and large size. A conventional AFE device uses more analog-to-digital converters, amplifiers, and filters for individual electrodes. Our proposed MUX-based AFE design requires fewer components than a conventional AFE device, reducing power consumption and area. It includes a single-ended differential feedback operational transconductance amplifier (OTA) and n-pass MUX-based AFE circuits which are related to the emergence of low power, low area, and low cost AFE-integrated chips that are required for wearable biomedical applications. The proposed 6T n-pass multiplexer measures a gain of −68 dB across a frequency range of 100 kHz with a 136.5 nW power consumption and a delay of 0.07 ns. The design layout area is approximately 9.8 µm^2^ and uses 45 nm complementary metal oxide semiconductor (CMOS) technology. Additionally, the proposed single-ended differential OTA has an obtained input referred noise of 0.014 µV_rms_, and a gain of −5.5 dB, while the design layout area is about 2 µm^2^ and was designed with the help of the Cadence Virtuoso layout design tool.

## 1. Introduction

The healthcare systems of all nations must adapt to the difficulties posed by new diseases, as well as dietary habits, way of life changes, environmental changes, and other factors. Researchers face novel and fascinating challenges due to the consumer healthcare market’s requirements, which are influenced by statistically solid characteristics of human populations (such as age or income) [1,2]. A World Health Organization (WHO) report reports that the leading cause of death globally are cardiovascular diseases (CVDs), which are expected to claim 17.90 million lives annually [3].

The Stroke and Heart Disease Statistics Update Fact Sheet provides information about the global prevalence of heart disease, stroke, and other ailments [4]. Strokes and heart attacks account for around 32 of every 100 deaths from CVD worldwide. In 2030, 1.56 billion people will have high blood pressure (BP), sometimes known as hypertension. Early detection and management of medical problems can significantly impact healthcare costs and survival rates. Medical professionals can now identify ailments earlier than ever due to biomedical technologies. They can now offer more individualized care and lower the possibility of difficulties during perfusion. It is possible to identify risks and treat medical diseases more quickly and effectively using wearables [5,6,7,8].

Rapid progress in the field of miniaturization within the electronic industry has made it possible to create more adaptive, reliable, and wearable devices. This might cause a global change in health systems toward the continuous monitoring of body health conditions [9]. Biomedical devices can be categorized as wearable and implantable therapeutic devices [10]. A WMD is a self-contained, non-invasive object that carries out a specific therapeutic or diagnostic purpose [11]. More research on WMDs was conducted between 2003 and 2023, and our literature analysis shows that this sector is proliferating. The use of wearable medical technology has vastly improved over the past decade, especially in the remote monitoring of people [12].

A WMD is a risk-detection device that must be small, light, and comfortable [13]. The three major industry sectors for the wearable market have been projected to be consumer electronics, defense, and health care. Smartwatches and bands are the most popular wearables on the market, according to statistics from the [14] Vandrico wearable index. This survey shows that 54% of wearables are functional, fashionable, wrist-mounted devices that consume a minimal amount of power among.

**Figure 1:**
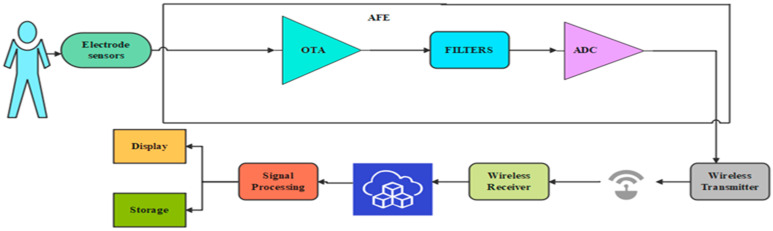
General block diagram of WMD.

An essential part of a WMD is shown in Figure 1. This wrist-worn device measures all biological signals with a 2 mW power consumption and a 1.2 V power supply [15]. With the help of wearable sensors, abnormal signals can be detected quickly, and medical aid can be provided promptly [16]. The constant electrode or sensor placement dramatically depends on the area where the biological signal is gathered from the skin surface [17]. An AFE primarily consists of amplifiers, filters, and analog-to-digital converter (ADC) blocks. Input voltages from electrode sensors are detected, amplified, filtered, and pre-processed by the amplifier and filter blocks. The output voltage is then converted into a digital signal by an ADC, which then transmits the signal to a transmitter [18]. A smartphone or comparable device receives the transmitted signal for real-time viewing. The data will then be sent to the cloud for additional processing and reporting to nearby physicians. 

The following discussion will be focused on previous research that was published regarding each block and how their designs were put into implementation. A 0.18 µW CMOS fabrication method was used to create an AFE—CMOS circuit proposed by Zhu et al. While occupying 1 mm^2^, it only consumes 1.3 µW at 0.5 V [19]. Kong [20] has proposed a pseudo-differential capacitive MEMS accelerometer AFE design using 0.18 µm CMOS technology. Output modulation moves the low-frequency capacitance sensor output from 2 to 12 kHz to the high frequency 800 kHz range. In turn, it receives a CMRR of 95 dB and a 0.5–250 Hz span. The main part of our proposed WMD is an MUX-based AFE design with a 4 × 1 6 T n-pass multiplexer, a single-ended differential feedback OTA, and a low-power CMOS Schmitt trigger. Simulations undertaken with the Cadence Virtuoso tool show that the proposed AFE design, which uses 45 nm technology with a span of 100 kHz, uses less power and takes up less space in an integrated IC design.

Previous research has shown that a healthy resting heart rate should range between 50 and 70 beats per minute [21]. Electrocardiography (ECG) electrodes convert beats into voltage, which then produce extremely weak signals ranging from 0.5 mV to 5.0 mV [22]. A normal electromyography (EMG) signal typically has an amplitude of 10 mV and a bandwidth of 0–500 Hz, with the dominant frequency range being 50–150 Hz [23]. Hypothermia is a drop in body temperature below 95°F. Precision temperature-to-voltage converters, or TC107/TC1047A sensors, can precisely measure temperatures between −40 °C and +125 °C [24]. According to the microchip technology datasheet, the output range is 100 mV at −40 °C, 500 mV at 0 °C, and 750 mV at +25 °C. Nakata et al. [25] have proposed a wearable, flexible, multipurpose healthcare device incorporating an ISFET chemical sensor for simultaneous sweat PH and skin temperature monitoring. Nyein et al. [26] have proposed that a given biological fluid may have a pH level of 3–8, with potentials ranging from 285.6 to 309.8 mV and sensitivities able to determine concentration changes ranging from 60.0 to 65.4 mV/decade.

Dua et al. [27] have designed a 45 nm CMOS MUX for high-speed applications with a supply of 1 to 3 volts, a frequency of 1 MHz, and a power consumption of 92.22 to 146.7 pW. Sharma and Akashe [28] designed multiplexers using XOR-based transmission gate techniques for low-power circuit applications. They compared their designs with different supply voltages from 0.8 to 5 volts and inferred that the power consumption varied from 0.152 µW to 0.829 mW. Kumar et al. [29] have proposed an nMOS 4X1 MUX using dynamic threshold MOS (DTMOS) technology with a 1 V supply and a 42.7 µW power consumption for low-power wearable device applications. 

Diab et al. [30] have developed a 130 nm CMOS AFE with adjustable low-pass and high-pass corner frequencies and a mid-band gain of 31 to 52 dB. This is used in low-noise biomedical applications. Then, Singh et al. [31] suggested an OTA-based readout front-end with a power consumption of 145.9 nW and a CMRR of 71.39 dB as an alternative to the current-mode method. This study defines a fundamental and unique structure while considering electrical characteristics such as DC gain and linearity. Later, Gifta et al. [32] proposed an ultra-low-power bulk-driven OTA circuit for biological applications. In this circuit, the double recycling technique was applied with a 0.4 V supply voltage, which increased the DC gain and slew rate. 

The Schmitt trigger, a 1-bit digitizer with pulse hysteresis, is one of the most vital parts of the ADC because it digitalizes the amplified biological signal. Parveen et al. [33] have claimed that current sink and current source inverters be used to build Schmitt trigger circuits for low-power and high-speed applications. The main advantage of the recommended circuits, which make use of a 180 nm CMOS model with cadence and a supply rail voltage of +3 V, is that they have stable output amplitudes in the 0° to 100 °C range. Pahlavan et al. [34] have suggested a dual-band Schmitt trigger suitable for multiband and high-performance applications. The power consumption and delay of the proposed delay element are 103–581 ps and 4.23–1.04 mW for CMOS 180 nm standard technology, respectively.

**Figure 2:**
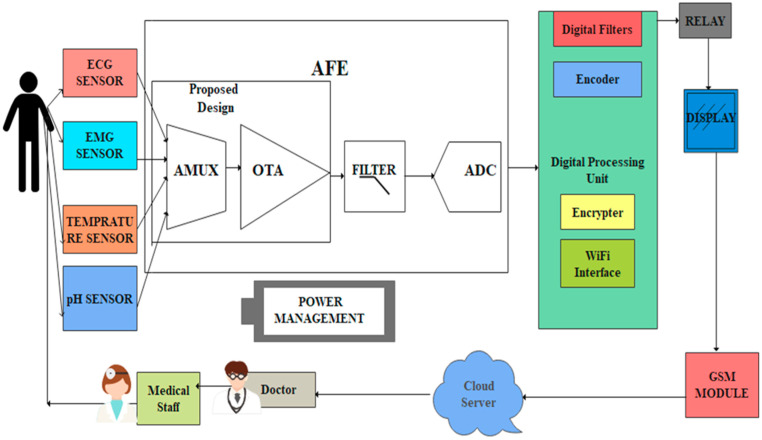
The proposed architecture of WMD with AFE.

The proposed WMD architecture has been designed by sharing amplifiers and filters across a large number of electrodes using the rapid multiplexing technique, as shown in Figure 2. 

Biophysical symptoms, including pain, fatigue, and heart problems, can all be better diagnosed with the use of EMG, ECG, pH, and temperature readings. The simultaneous collection of multiple signals simplifies the identification of the root cause of stress- or discomfort-related illnesses. The AFE includes a 4 × 1 MUX that receives bio-information from EMG, ECG, pH values, and temperature sensors. AFE is essential in WMDs for signal amplification and for identifying human abnormalities. 

Each electrode in a conventional AFE must have its own amplifier, filter, and ADC converter, which creates a variety of design challenges, such as the large active area of resistors, complexity, scalability, and power consumption [35,36,37]. The MUX-based AFE is suggested in this study as a solution to these problems. An analog 6T n-pass time division multiplexer, a single-ended fully differential OTA, and a low-power Schmitt trigger have all been designed for the proposed MUX-based AFE. The performance of each component has been evaluated using CMOS technologies.

Individuals are monitored via a wearable device, which must be worn at all times. This detects deviations from the typical pre-set values and alerts the end user so they can take the right action. A crew and medical professionals regularly observe the patients using the Internet of Things (IoT) in their current circumstances. The solution might be a continuous monitoring system that guards against casualties while urging the crew to seek medical attention in emergencies.

## 2. Materials and Methods

### 2.1. Design a 4 × 1 Multiplexer Using n-Pass Time Division Techniques 

**Figure 3:**
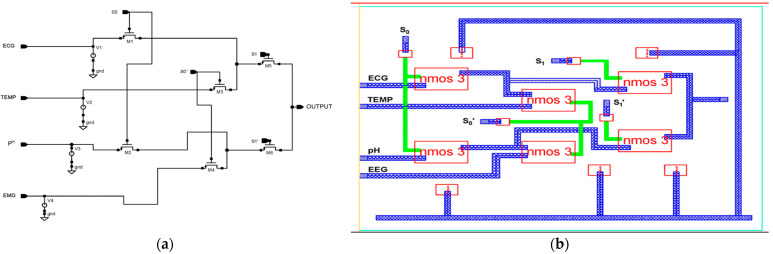
Proposed 45 nm low power n-pass 4 × 1 MUX. (**a**) Schematic diagram and (**b**) layout structure.

The proposed 4 × 1 n-pass time division analog multiplexer design is depicted in Figure 3a using 45 nm CMOS technology. The 4 × 1 time division multiplexing (TDM) technique sends four distinct signals across a signal line using synchronized switches, which are transmitted sequentially in time. The multiplexer has been designed using low-power pass transistor logic (LPPTL), which requires only six transistors. The source/drain terminals of the n-pass transistors are connected to the inputs from electrodes with dimensions of L = 45 nm and W = 120 nm. Figure 3b illustrates the physical layout structure of a 4 × 1 n-pass MUX.

The sampled input values are taken from the sensors: 10 mV for EMG, 63.3 mV for pH, 100 mV for temperature, and 5.0 mV for ECG. The truth table for the 4 × 1 n-pass time division analog multiplexer used in the circuit diagram is presented in Table 1. The selection lines S1, and S0 select which inputs should be relayed to the output. Every input has a potential generated by Equation (1) [38].

Output = S_1′_S_0′_ EMG + S_1′_S_0_ TEMP + S_1_S_0′_ pH Value + S_1_S_0_ ECG. (1)

The pass transistor logic reduces the number of transistors needed to implement logic by allowing the primary inputs drive both the gate and source-drain terminals. It operates 88% faster, reduces area by 70%, and consumes 81.9% less energy than fully complementary MOS logic.

### 2.2. Design of Low Power Differential Feedback OTA

**Figure 4:**
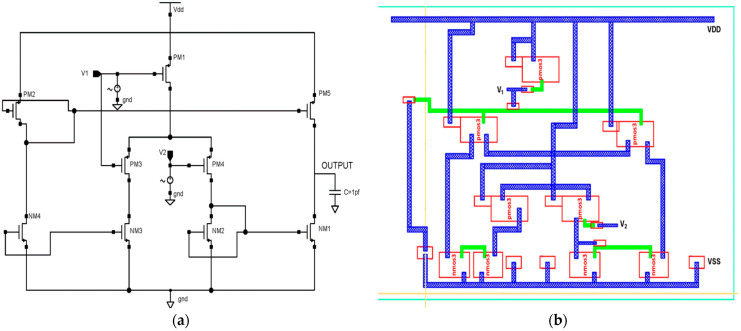
Proposed 45 nm low power differential feedback OTA design. (**a**) Schematic diagram and (**b**) layout structure.

In this section, a single-stage differential positive feedback OTA has been designed using source degeneration techniques, as shown in Figure 4a. When significant signals are applied, the circuit converts a differential pair input voltage into the current as an output. The bias circuit generates an external voltage that serves as a reference voltage and drives the entire circuitry. We have drawn the physical layout structure, depicted in Figure 4b, with dimensions of 14.4 μm × 14.45 μm.

PMOS transistors are used in the input stage because of their low flicker noise output. The transistors PM1–PM4 make up the differential gain stage, the input’s first stage. The gate terminals of the transistors PM1 and PM2 are correspondingly connected with the differential voltage signal. This OTA is built with two voltages, Vin+ and Vin-, coupled to NM2 and NM3, respectively. Transistors PM1 and PM2 are coupled back-to-back in the first stage. The gates of each CMOS transistor are electrically connected with this technology. 

The differential amplifier characteristics of transistors PM3 and NM4 allow for reduced power usage. While NMOS transistors, such as NM1, NM2, and NM4 operate in the sub-threshold zone and need less power, PMOS transistors such as PM1, PM2, and PM3 operate in the linear area. 

Total charge

Q = WL C _ox_ [(V _GS_ − V _T H_) − 0.5 V _DS_]. (2)

where current I = Q/τ, τ is transient time.

The current equation for CMOS is

I_D_ = µC_ox_W/L [(V_GS_ − V _T H_) V_DS_ − V^2^_DS_/2] (3)

The total charge Q is expressed in Equation (2) [39], where W/L represents the transistors’ width-to-length ratios, I_D_ is the drain current, µ is the MOS transistor’s mobility, C_ox_ is the oxide layer’s capacitance, V_GS_ is the gate-to-source voltage, V_TH_ is the threshold voltage, and V_DS_ is the drain-to-source voltage. To create a linear transconductor with a broad input operating range, the proposed design is subject to a four-NMOS transistor cell that gives a low transconductance path. In the design of the fully differential transconductor, two output currents, I_1_ and I_2_, are produced by the voltage-to-current converter (V–I). The output current Io is linearly proportional to the differential input voltage (V_ol1_ − V_ol2_), such that Io = I_2_ − I_1_ = G (V_ol1_ − V_ol2_), where G is the equivalent transconductance. Equation (3) [39] is used to design a low-power differential feedback OTA architecture and analyze the drain current (I_D_) using a 45 nm CMOS model with minimum transistor sizing. The NMOS and PMOS transistor sizing are given in Table 2 and Table 3.

A low-power differential feedback OTA design is developed using Equations (4) and (5) [36], and the drain current ID is evaluated using a 45 nm CMOS mode. The configurations of PMOS and NMOS are then sized in terms of length, width, finger, threshold, and SD metal width based on Table 2 and Table 3, mentioned above. It is possible to find the perfect transistor size for a given set of requirements by using optimization techniques. Transistor sizing is often required for a number of performances, including the transconductance parameter of the NMOS, PMOS (k_n_, k_p_) Equations (3) and (5), and the transconductance of the NMOS, PMOS (g_mn_, g_mp_) Equations (6) and (7) [40]. This is used to analyze the OTA’s power consumption, gain, frequency response, and linearity [41].

For NMOS, the transconductance parameter factor (k_n_) is:

k_n_ = µ_n_C_ox_(W/L); V_tn_ > 0; V_DS_ ≥ 0; V_ov_ = V_GS_ − V_tn_ (4)

Transconductance of NMOS (g_mn_) is

g_mn_ = k_n_V_ov_ (5)

With PMOS, the formula for the PMOS transconductance parameter factor (k_p_) is:

k_p_ = µ_p_C_ox_(W/L); V_tp_ < 0; V_SD_ ≥ 0; V_ov_ = V_SG_ − |V_tp_| (6)

The PMOS transconductance (g_mp_) is given by

g_mp_ = k_p_.V_ov_ (7)

where µ_n_ is the mobility of the NMOS transistor, µ_p_ is the mobility of the PMOS transistor, C_ox_ is the capacitance of the oxide layer, V_GS_ is the gate-to-source voltage, W/L is the width-to-length ratio of the transistors, V_tn_ is the threshold voltage of the NMOS transistor, V_tp_ is the threshold voltage of the PMOS transistor, and V_DS_ is the voltage from the drain to the source. V_ov_ is a voltage for overdrive.

The transistors NM1–NM4 make up the differential gain stage, which is the input’s first stage. The gate terminals of the transistors PM1 and PM4 are correspondingly connected with the differential voltage signal. PM2, PM3, and PM5 give a balanced differential gain to the entire circuit with a high driving capacitive. The proposed OTA is a voltage-controlled current source, which provides the benefit of a significantly lower transistor count. This is the most straightforward and successful OTA for wearable medical applications. 

### 2.3. Design of Low-Power CMOS Schmitt Trigger

**Figure 5:**
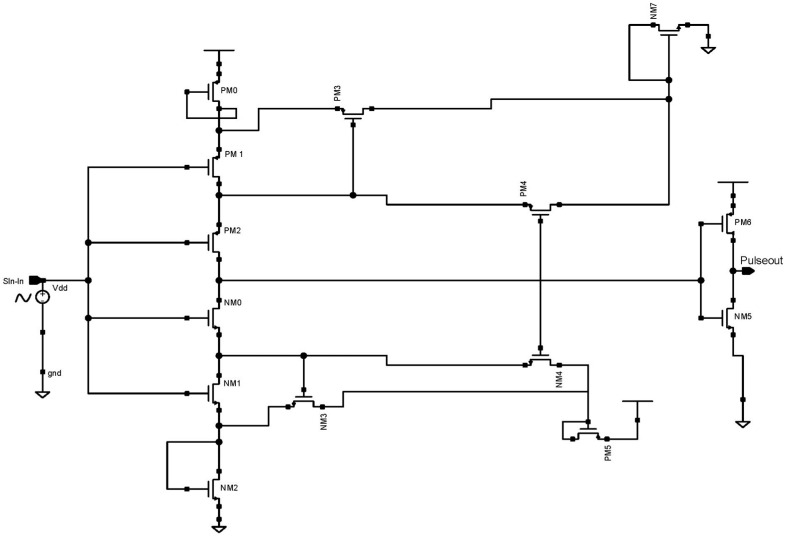
Schematic diagram of 45 nm proposed CMOS Schmitt trigger.

Figure 5 depicts a CMOS Schmitt trigger that digitizes an analog input signal at a low-level supply voltage. Self-bias transistors PM0, NM7, NM2, and PM5 lower the effective supply voltage level. The transistors PM3 and NM3’s width and length can be changed to create pulse hysteresis. When the input voltage is low, NM1 is disabled and PM1, PM2, and NM0 currents are almost zero. The output begins to decline as soon as NM0 is turned on.

Before NM0 is turned on, the currents of NM4 and NM1 are equal. We considered the use of a low-power CMOS Schmitt trigger and a low-level supply voltage was used to digitize the input-modulated signal in this device. Transistors that self-bias were utilized to lower the supply voltage’s level. A voltage inverter was utilized at the output to balance the voltage swing. The circuit was simulated using 45 nm CMOS technology at a 100 Hz input sine wave. According to the simulation results, the suggested Schmitt trigger used a 0.8 V supply and a standard 45 nm PMOS and NMOS size to consume 1.22 nW power.

### 2.4. Design of AFE IC

This patient-monitoring IC is an AFE for recording numerous vital human body parameters through the skin. This AFE-on-a-chip enables clinical-grade vital sign measurement and bio-signal processing for patient health assessment, focusing on manufacturers of compact, battery-operated, constantly wearable devices.

**Figure 6:**
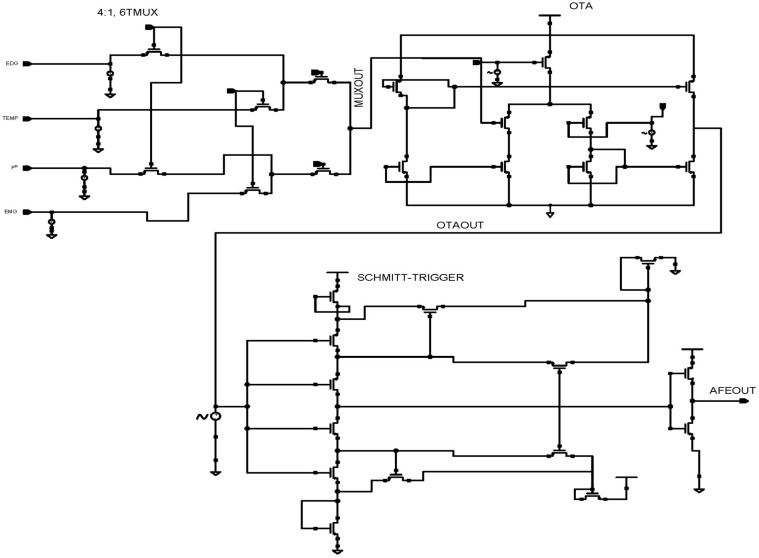
Schematic diagram of the 45 nm proposed AFE design for WMD.

Figure 6 illustrates the proposed AFE for WMD applications, which consists of the 4 × 1 time division multiplexer, differential OTA, and low-power Schmitt trigger. Vital human body signals, i.e., ECG, EMG, pH values, and temperature, are combined by the 4 × 1 time division multiplexer, which also uses a low-power Schmitt-trigger ADC to digitize current signals from OTA. In total, the proposed AFE consists of 29 transistors and consumes 2.62 pW power from a 0.8 V supply voltage. Cadence Virtuoso tools, styled as cādence, is an American international computational software firm headquartered in San Jose, California. It was created in 1988 through the merging of SDA Systems and ECAD, Inc. These tools offer a robust design environment and were utilised in the present research for analysis purposes. The Cadence Licence Manager was successfully installed and executed on a Linux-based personal computer operating on the Ubuntu distribution. 

## 3. Results and Discussion 

### 3.1. Proposed 4 × 1 Multiplexer Simulation Results and Comparison Analysis

#### 3.1.1. Transient Response

**Figure 7:**
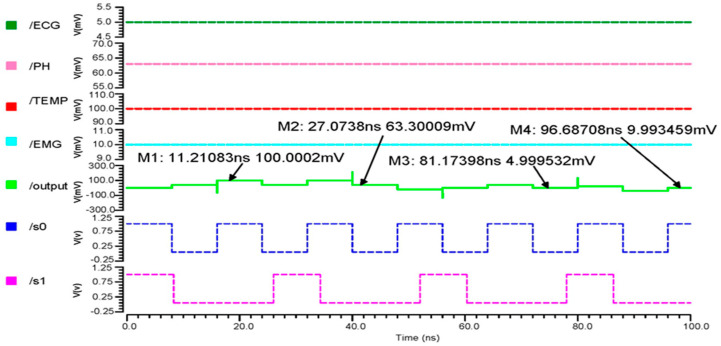
The 4 × 1 MUX transient response output wave form.

In our proposed design, the output produced the least possible delay due to time division multiplexing techniques. The combination of inputs at the selection lines S_0_ and S_1_ determines the multiplexer’s output. The sensors’ sampled input values were 10 mV for EMG, 63.3 mV for pH, 100 mV for temperature, and 5.0 mV for ECG. The 4 × 1 MUX transient response output wave form is seen in Figure 7. Marker M1 indicates an 11.21083 ns output as 100.0002 mV, which is the temperature output when the pulse input S_0_ = 1, S_1_ = 0 with the least error 0.0002, indicating our proposed circuit runs more accurately.

#### 3.1.2. AC Analysis

**Figure 8:**
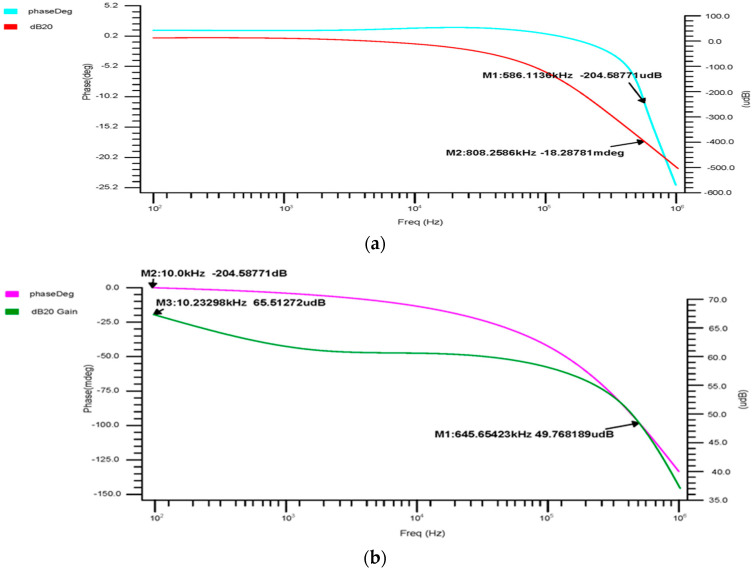

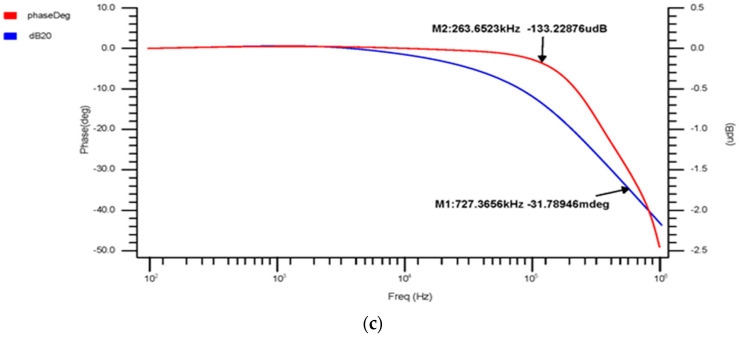
4 × 1 MUX phase-versus-gain AC response for the (**a**) 180 nm technology, (**b**) 90 nm technology and (**c**) 45 nm technology.

The 180 nm 4 × 1 multiplexer’s phase-versus-gain output curve, shown in Figure 8a, indicates that the cutoff frequency is 586.1 kHz and the gain is −204.9 mdB, with a 3 dB phase margin of −16.68°. Figure 8b shows the 90 nm 4 × 1 multiplexer output curve, with a cutoff frequency of 203.652 kHz, a gain of −133.92 mdB, and a 3 dB phase margin of −31.76°. The 45 nm 4 × 1 multiplexer’s phase-versus-gain output curve is shown in Figure 8c. The above results show that the gain margin (GM) increases the stability of the 4 × 1, which is suitable for accurately gathering and analyzing the bio-signal from the sensors.

#### 3.1.3. Comparison Results of 4 × 1 MUX

This research analyzed in detail the low-power 4x1-MUX n-MOS 6T pass transistor logic. Table 4 demonstrates that 45 nm CMOS-based 6T-n-pass logic requires less power and physical space than other technologies. Table 5 provides the parameter analysis based on simulations undertaken with Cadence Virtuoso, such as area, bandwidth, phase, and gain margin, for 180, 90, and 45 nm technologies. However, the number of transistors in our suggested design is decreased to 6 due to the low-power n-pass- MOS logic technique. Using 45 nm CMOS, a latency of almost 0.07 ns and low power consumption of 136.5 nW have been achieved.

### 3.2. Proposed OTA Simulation Results and Comparison Analysis

#### 3.2.1. Transient Analysis

**Figure 9:**
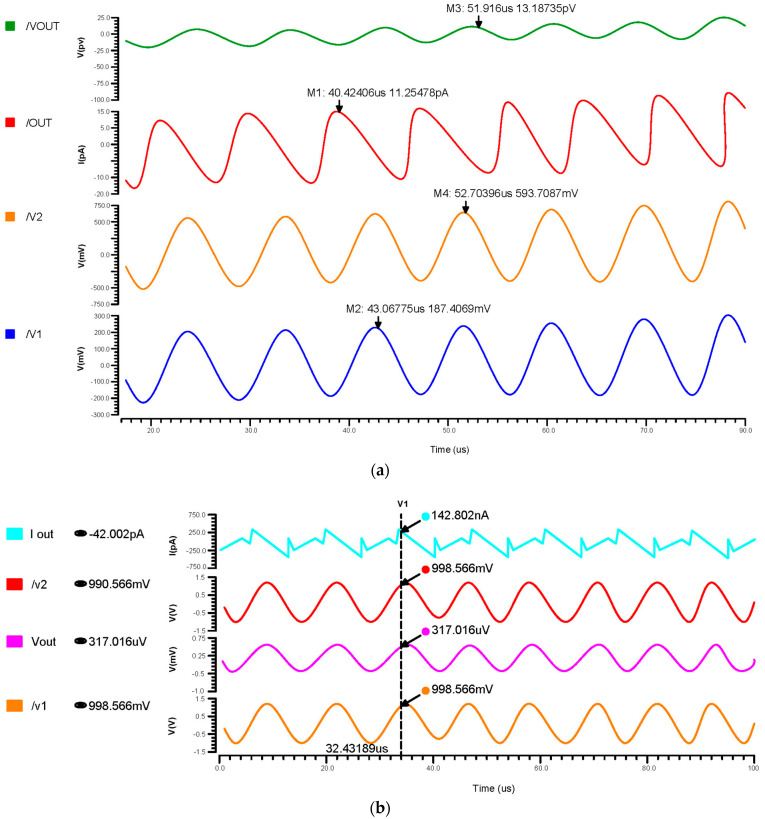

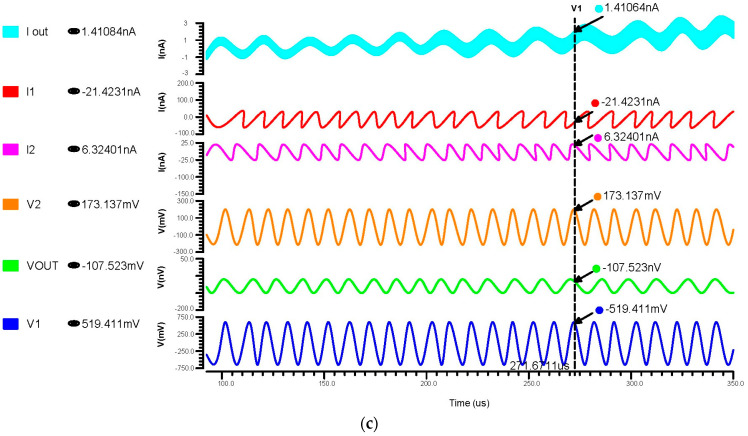
Transient response of differential OTA using (**a**) 180 nm technology, (**b**) 90 nm technology and (**c**) 45 nm technology.

We have undertaken transient analysis for differential feedback OTA, using 180 nm, 90 nm, and 45 nm technologies as shown in Figure 9a–c, respectively. These consumed 130 nW for the 180 nm, 1.5 nW for the 90 nm, and 1.43 nW for the 45 nm technology. A two-stage operational transconductance amplifier based on 45 nm CMOS technology has been designed and analyzed with a supply voltage of 0.8 V. We inferred that lowering the nm technology produced a low power consumption.

#### 3.2.2. AC Analysis

**Figure 10:**
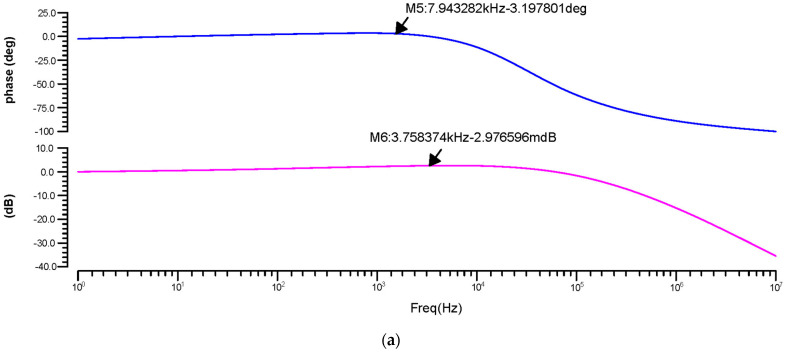

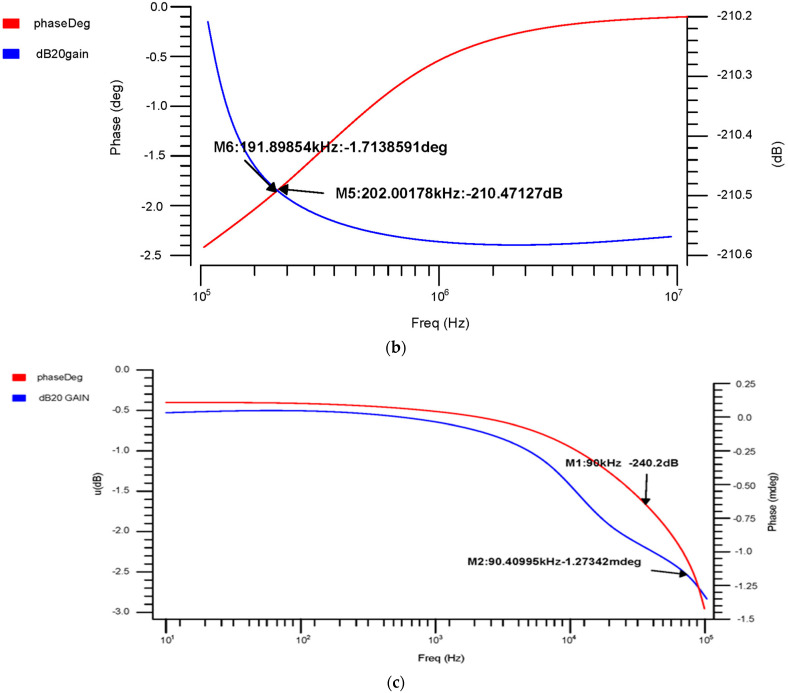
Differential OTA phase-versus-gain AC response for the (**a**) 180 nm technology, (**b**) 90 nm technology and (**c**) 45 nm technology.

The phase and transient reaction are also important components of an amplifier, as the AC frequency response is used to determine the gain and bandwidth. The gain-versus-phase response was analyzed and the gain value was −59.07 dB at 43.19 kHz for 180 nm, 2.97 mdB at 3.75 kHz for 90 nm, and −1.23 dB at 90.40 kHz for 45 nm, as shown in Figure 10a–c.

#### 3.2.3. Noise Analysis of Proposed OTA

**Figure 11:**
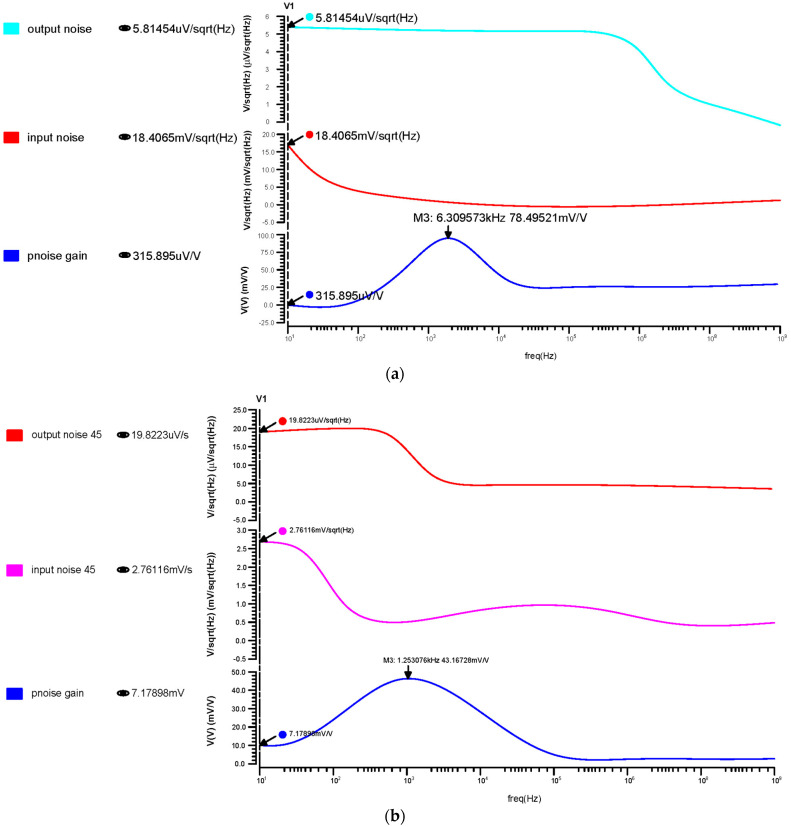

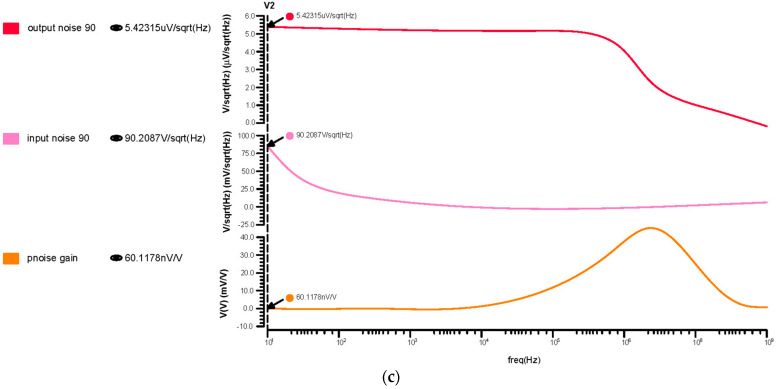
Differential OTA input, output, and transfer noise response for the (**a**) 180 nm technology, (**b**) 90 nm technology and (**c**) 45 nm technology.

Each component’s noise contribution is estimated at the output node over the chosen frequency range. The input referred noise has been calculated as 2.05 µmV_rms_ for 180 nm, 0.093 µmV_rms_ for 90 nm, and 14.76 µmV_rms_ for 45 nm, as shown in Figure 11. From these results, the 45 nm technology offered a low noise that brings a noise-free signal amplification for biological applications.

#### 3.2.4. Comparison Results of OTA

From Table 6, we inferred that the proposed design had a low power consumption of 1.43 nW in the 45 nm CMOS technology. Comparing our work historic values revealed that it reached up to 0.014 µV_rms_ and that the noise efficiency factor is 1.6.

### 3.3. Proposed AFE Simulation Results and Comparison Analysis

**Figure 12:**
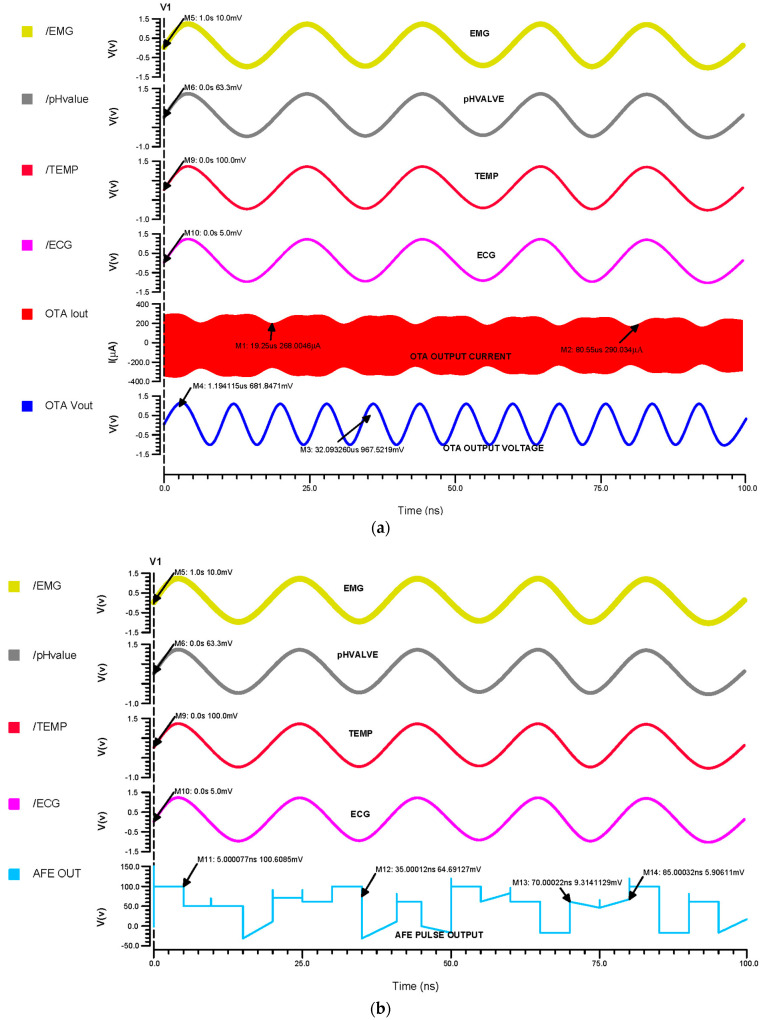

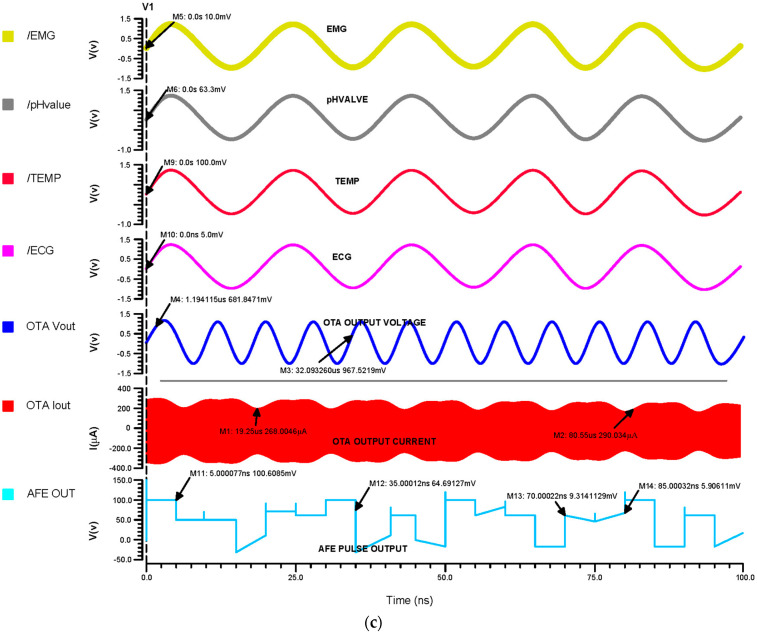
The (**a**) 45 nm OTA transient analysis, (**b**) 45 nm Schmitt trigger transient analysis and (**c**) 45 nm AFE transient analysis.

Figure 12a indicates that the inputs from the sensors range in voltage from 5 mV to 100 mV, which differential OTA amplifies to about 680 mV, and that their currents are then in the range of about 300 µA, Figure 12b shows that the input of the Schmitt trigger analog current signal is converted to a voltage pulse signal. The Schmitt trigger output ranges from 5 mV to 110 mV, depending on the inputs at different times. Using a differential OTA circuit, an AFE device is able to read analog data from sensors for the ECG, EMG, pH value, and temperature before converting them to current. A Schmitt trigger circuit then changes the current input to pulses by time variation. This is illustrated in Figure 12c.

As inferred from Table 7, the proposed AFE design demonstrated a low power consumption of 2.62 pW for the 45 nm CMOS technology. The individual power supplies for the MUX, OTA, and Schmitt trigger circuits are consolidated into a single source for all of the different circuits in order to drastically minimize the power needed for the integrated AFE circuit.

## 4. Conclusions

This research shows the design and development of a multiplexer-based AFE for WMD using CMOS techniques. The main part of the proposed WMD is an MUX-based AFE design made up of a 4 × 1 6T n-pass multiplexer, a single-ended differential feedback OTA, and a low-power CMOS Schmitt trigger. According to the results of simulations performed using the Cadence Virtuoso tool, the proposed AFE design, which uses 45 nm technology, only consumes 2.62 pW and occupies less space in an integrated IC design. In addition, the simulation result of the 6T n-pass multiplexer has a gain of −68 dB across a frequency range of 100 kHz and consumes 136.5 nW of power. This is used to combine all of the biosignals, including ECG, EMG, pH, and temperature, for the proposed AFE. The OTA plays a vital role in the AFE device. The suggested circuit for the single-ended differential OTA exhibits a power consumption of 1.43 nW, a gain of −5.5 dB, and a noise factor of 1.6. This circuit enhances the amplification of biosignals, and thus surpasses the previous research in terms of its scope and results. Wearable continuous person monitoring systems may benefit from the usage of MUX-based AFE in our research, which will be used to declare long-lasting battery lifespan for consumer electronics products with reliable, compact real-time monitoring features.

## Figures and Tables

**Table 1:** Proposed 4 × 1 MUX truth table.

Sel Lines		Inputs from the Various Electrodes				Outputs
S_1_	S_0_	ECG	PH VALUE	TEMP	EMG	Outputs
0	0	0	0	0	10 mV	10 mV
0	1	0	0	100 mV	0	100 mV
1	0	0	63.3 mV	0	0	63.3 mV
1	1	5.0 mV	0	0	0	5.0 mV

**Table 2:** PMOS sizing.

PMOS	L	W	Finger Width	Threshold	SD Metal Width
PM1	1 µm	2 µm	2 µm	800 nm	400 nm
PM2	1 µm	20 µm	20 µm	800 nm	400 nm
PM3	1 µm	2 µm	2 µm	800 nm	400 nm
PM4	1 µm	30 µm	30 µm	800 nm	400 nm
PM5	1 µm	30 µm	30 µm	800 nm	400 nm

**Table 3:** NMOS sizing.

NMOS	L	W	Finger Width	Threshold	SD Metal Width
NM1	5 µm	20 µm	20 µm	800 nm	400 nm
NM2	1 µm	20 µm	20 µm	800 nm	400 nm
NM3	1 µm	20 µm	20 µm	800 nm	400 nm
NM4	1 µm	20 µm	20 µm	800 nm	400 nm

**Table 4:** Comparison analysis of 4 × 1 MUX using different technologies from previous research.

Work Done	Technique Used	Transistor Count	Technology Used (nm)	Power Consumption (W)	Delay (ns)
[28]	Static CMOS logic	36	45	7550n	0.588
[29]	TGL logic	18	45	312.8n	0.075
[42]	nMOS logic	6	45	234.5n	0.0483
This paper	nMOS–pass transistor logic	6	180	337.9n	0.603
This paper	nMOS–pass transistor logic	6	90	223.7n	0.598
This paper	nMOS–pass transistor logic	6	45	136.5n	0.07

**Table 5:** Parameter analysis of various CMOS technologies.

Work Done	Technique Used	Transistor Count	Technology Used (nm)	V_rms_ (mV)	I_rms_ (µA)	Phase Margin (mdeg)	Gain Margin (mdB)	Band width (kHz)	Area (µm^2^)
This paper	N-pass Transistor Logic	6	180	12.7	176.6	16.68	−204	586.10	116.64
This paper	N-pass tran-sistor logic	6	90	23.92	14.12	31.769	−133	203.65	0103.4
This paper	N-pass tran-sistor logic	6	45	61.90	2.205	60.25	−68	141.8	009.8

**Table 6:** Comparison of OTA power with different technology.

Year/Ref. No.	2003/ [43]	2007/ [44]	2010/ [45]	2011/ [5]	2012/ [46]	2014/ [47]	2016/ [48]	2017/ [39]	2017/ [49]	2018/ [50]	2019/ [51]	2019/ [40]	2020/ [31]	2021/ [52]	2023/ [53]	This Paper		
CMOS/nm TECH	150	180	180	90	18	90	180	130	180	180	180	45	180	180	180	180	90	45
Application	E	E	E	E	E	E	E	E	E	E	E	E	E	E	E	W	W	W
	E	C	C	E	C	C	E	E	C	E	C	C	C	C	C	M	M	M
	G	G	G	G	G	G	G	G	G	G	G	G	G	G	G	D	D	D
Power Consumption (w)	0.9 µ	15 n	110 µ	3.6 µ	7.24	2.6 µ	2.2 µ	216 n	15 n	593 n	1.55 µ	11.1 µ	5 µ	1.9 n	383 n	130 n	1.5 n	1.43 n
Supply Voltage (V)	2.5	1	1	1.2	1	0.5	1	0.4	0.5	1.8	1	1	1	±0.2	0.4	1	0.8	0.8
Phase Margin (µdeg)	-	−71	-	−49	-	−52	-	-	-	-	-	-	−40	-	-	−11	−25	−22
Gain (dB)	-	−62	-	−10	-	-	−6.1	−80	−10.5	−10	−10	−10	-	-	-49	−59	−6.1	−5.5
Bandwidth (kHz)	100	100	250	-	-	100	250	50	100	240	2.4	-	100	250	50	100	100	100
Area in (mm^2^)	0.16	0.201	0.13	0.45	0.2	0.12	-	0.189	0.12	0.09	0.18	0.016	-	0.018	-	0.0154	0.0095	0.002
Input referred noise (µV_rms_)	2.2	3.6	-	2.48	0.59	6.27	3.2	1.69	1.113	5.79	2.05	0.65	3.15	3.55	-	2.05	0.093	0.014
Noise efficiency factor (NEF)	-	-	-	3.09	-	0.64	1.86	-	-	3.16	2.26	-	-	-	-	3.2	2.4	1.6

**Table 7:** AFE power comparison using various technologies.

Nano Meter Technology	MUX P_rms_ (nW)	OTA P_rms_ (nW)	ADC (Schmitt Trigger) P_rms_ (nW)	Total (MUX + OTA + ADC) P_rms_ (nW)	AFE IC P_rms_ (nW)
180 nm	2234	13,000	17,950	18,220	9.988
90 nm	337.9	1.5	2.08	349.8	0.236
45 nm	136.5	1.43	1.22	139.15	0.026

## Data Availability

The article contains all the necessary data to support the results, and there is no need for any more source data.
